# Trajectories of body mass index, from adolescence to older adulthood, and pancreatic cancer risk; a population-based case–control study in Ontario, Canada

**DOI:** 10.1007/s10552-019-01197-9

**Published:** 2019-06-22

**Authors:** Vanessa De Rubeis, Michelle Cotterchio, Brendan T. Smith, Lauren E. Griffith, Ayelet Borgida, Steven Gallinger, Sean Cleary, Laura N. Anderson

**Affiliations:** 1 Department of Health Research Methods, Evidence, and Impact, McMaster University, 1280 Main Street West, Hamilton, ON L8S4L8 Canada; 2 Prevention and Cancer Control, Cancer Care Ontario, Toronto, ON Canada; 3 Public Health Ontario, Toronto, ON Canada; 4 Dalla Lana School of Public Health, University of Toronto, Toronto, ON Canada; 5 Division of General Surgery, Toronto General Hospital, Toronto, ON Canada; 6 Lunenfeld-Tanenbaum Research Institute, Mount Sinai Hospital, Toronto, ON Canada; 7 Department of Surgery, University Health Network, University of Toronto, Toronto, ON Canada; 8 Division of Hepatobiliary and Pancreas Surgery, Mayo Clinic, Rochester, MN USA; 9 Child Health Evaluative Sciences, The Hospital for Sick Children Research Institute, Toronto, ON Canada

**Keywords:** Body mass index, Obesity, Trajectory, Life-course, Pancreatic cancer

## Abstract

**Purpose:**

Pancreatic cancer has the highest fatality rate of all cancers. Adulthood obesity is an established risk factor for pancreatic cancer; however, life-course obesity is not well understood. The aim of this study was to evaluate the association between body mass index (BMI) trajectories throughout the life-course and pancreatic cancer risk.

**Methods:**

A population-based case–control study was conducted (2011–2013) in Ontario, Canada. Cases were recruited from the Ontario pancreas cancer study (*n *= 310) and controls from the Ontario cancer risk factor study (*n* = 1258). Questionnaires captured self-reported height and weight at four timepoints (adolescence, 20 s, 30–40 s, 50–60 s). BMI trajectories were identified using latent class growth mixture modeling. Odds ratios (OR) and 95% confidence intervals (CI) were estimated from multivariable logistic regression.

**Results:**

Five BMI trajectories were identified: stable-normal weight (38.9%), progressively overweight (42.2%), persistent overweight (12.6%), progressive obesity (4.2%), and persistent obesity (2.1%). The persistent overweight (OR = 1.55; 95% CI 1.02, 2.39) and progressive obesity trajectories (OR = 1.49; 95% CI 0.77, 2.87) compared to stable-normal weight were associated with increased odds of pancreatic cancer. When BMI was evaluated separately the strongest associations with pancreatic cancer emerged in young and mid-adulthood.

**Conclusion:**

BMI trajectories characterized by overweight in early adulthood were associated with increased pancreatic cancer risk suggesting a life-course approach to disease risk.

**Electronic supplementary material:**

The online version of this article (10.1007/s10552-019-01197-9) contains supplementary material, which is available to authorized users.

## Introduction

Pancreatic cancer has the highest fatality rate of all cancers, with a 5-year survival rate of 8% [1]. The age-standardized incidence rate has remained relatively stable, with an annual percent decrease of only 0.1% per year since 1992 [2]. Globally, pancreatic cancer is the 12th most common cancer [3]. Primary prevention is important since 80% pancreatic cancer cases are diagnosed at an advanced stage due to difficulties associated with early detection, and no available screening programs [4]. Despite the high fatality rate, pancreatic cancer only accounts for approximately 3% of all cancer cases, thus prospective population-based studies of this disease are very difficult [5]. Some of the well-established risk factors for pancreatic cancer are non-modifiable, including older age, genetics, and family history [6]. The most well-established modifiable risk factors that have been identified are obesity and smoking [6–8].

Overweight and obesity in adults, most often defined as body mass index (BMI) > 25 kg/m^2^ [9], is strongly associated with pancreatic cancer [10–13] (RR: 1.43; 95% CI 1.19, 1.72) [14]. Globally, the prevalence of overweight and obesity has increased from 23.3 to 34.7% from 1978 to 2004 [15]. An association between obesity or high BMI in early adulthood and increased pancreatic cancer risk has been noted [10, 16–19]. However, few studies have evaluated the impact of BMI before age 18 [11, 20] or life-course trajectories of body size [21] on pancreatic cancer risk. Song et al., found that those who were consistently heavy, or increasingly heavy throughout their life compared to those who were lean-stable had an increased mortality due to pancreatic cancer (Males: HR: 1.15; 95% CI 0.79, 1.66; Females: HR: 2.15; 95% CI 1.49, 3.12) [22]. Understanding the impact of BMI over the life-course and the possible role of obesity in childhood in relation to pancreatic cancer risk is important given that the prevalence of overweight and obesity in children and adolescents has exceeded 30% [23, 24]. Further, children and adolescents with obesity have an increased risk of remaining obese throughout adulthood [25–28].

Despite the known association between obesity and pancreatic cancer risk, there is limited research assessing the impact of BMI trajectories across the life-course on pancreatic cancer risk. It is important to understand if there are sensitive periods across the life-course that differentially effect risk of pancreatic cancer. The primary objectives of this study were to identify trajectories of BMI over the life-course and to evaluate the association between these BMI trajectories from adolescence to older adulthood, and the risk of pancreatic cancer. The secondary objective of this project was to evaluate BMI during specific time periods of exposure in relation to odds of pancreatic cancer.

## Methods

### Study design

A population-based case–control study was conducted using cases from the Ontario pancreas cancer study (OPCS) and controls from the Ontario cancer risk factor study (OCRF) [29].

### Cases

Pancreatic cancer cases were recruited between 2011 and 2013 by the OPCS. The OPCS was one of seven study sites contributing data on genetic and epidemiologic factors among pancreatic cancer cases to the multidisciplinary pancreatic cancer genetic epidemiology (PACGENE) Consortium [7]. Cases were identified from the Ontario Cancer Registry rapid-case ascertainment system (electronic pathology reports). This is a population-based registry that obtains information for all cancer cases in Ontario through computerized probabilistic record linkage of data from pathology reports, regional cancer centers, hospital discharge and ambulatory care records, and Ontario death certificates. Men and women living in Ontario, who were diagnosed with pathologically confirmed adenocarcinoma of the pancreas or adenocarcinoma metastasis diagnosed by a physician (International Classification of Diseases for Oncology Third Edition codes C25.0–25.9, with 25.4 neuroendocrine pancreas excluded), were eligible for inclusion within the study. Cases with proxy respondents were excluded from this analysis. A total of 1310 cases of pancreatic cancer were diagnosed between February 2011 and January 2013, and of these, 314 were not mailed the study package (33 refused, 158 deceased or ineligible, and 123 unable to contact). From the 996 patients who were mailed the study package, a total of 414 (42%) returned completed questionnaires, but 40 cases with proxy respondents and 64 cases missing BMI data at 3 or more time periods were excluded, leaving 310 cases included in the analysis. 84% of cases had data for BMI at all four timepoints.

### Controls

Population-based controls were recruited through the OCRF in 2011. Controls were recruited using a modified random digit dialing procedure of households in Ontario and were frequency matched (3:1) on 5-year age and sex groups based on the expected distribution of cases. The OCRF identified 1995 eligible controls, 87% agreed to participate and were mailed the study package. The full epidemiologic questionnaire was completed by 1285 individuals (74%). 27 participants were excluded due to missing BMI data at three or more time periods, leaving 1258 controls included within the analysis. 87% of controls had data for BMI at all four timepoints.

### Research ethics

Research ethics approval for this study was obtained from the University of Toronto and Mount Sinai Hospital, Toronto, Canada. The protocol for the current secondary data analysis received research ethics approval from McMaster University, Hamilton, Canada.

### Data collection

Cases and controls who were eligible for participation were mailed a study package containing four self-administered questionnaires: epidemiology, family history, allergy, and food (short 55-item) [29]. If no response was received within 2 weeks of mailing the study package, participants were sent a reminder postcard, followed by telephone follow-up. If there was no response after 10 weeks, the participant was sent a second package.

### Measurement of body mass index

Cases and controls were asked to report their height “Approximately how tall are you (without shoes on)?”. Recall of body weight was asked for five distinct timepoints: adolescence, young adulthood (20 s), mid-adulthood (30–40 s), later adulthood (50–60 s) and current (for controls) or 1 year prior to cancer diagnosis (for cases). Self-reported recall of past weight has been noted to be a valid measure of true adiposity as it is highly correlated with prospectively collected data (pooled r = 0.91; 95% CI 0.91, 0.92) [30–32]. For the four earlier age periods, study participants were asked the same questions, “Approximately how much did you weigh as a teenager?” [33–37]. BMI (kg/m^2^) for each timepoint was calculated as weight in kilograms divided by height in meters squared. BMI cut-offs were determined using the World Health Organization guidelines, which considers a BMI greater than 25 kg/m^2^ overweight and a BMI over 30 kg/m^2^ obese [38]. We graphically inspected BMI at each timepoint to determine if there were any implausible values; none of the upper values were considered to be implausible (maximum BMI was 65), but there were three lower BMI values that were considered implausible and were changed to missing (*n*  = 3).

### Measurement of other variables

In general, exposures were assessed 2 years prior to cancer diagnoses for cases or 2 years earlier for controls. Variables associated with both the exposure (BMI) and the outcome (pancreatic cancer) were selected as potential confounders. These included: age, sex, education, race, family history of pancreatic cancer, cigarette smoking, alcohol intake, current physical activity (moderate and vigorous), and fruit, vegetable, and red meat intake. Diabetes and pancreatitis were identified as potential mediating variables and therefore were not included in the full model which adjusted for all confounders. Rather a third model was generated which included the full model plus diabetes and pancreatitis to determine the effect on BMI and pancreatic cancer risk. Data on covariates were collected through the epidemiology questionnaire. Cigarette smoking was categorized as never, current or former smoker. Alcohol was categorized as never, former, current light to moderate drinker (1–20 drinks/week), and current heavy drinker (> 21 drinks/week). Leisure and work physical activity from all sources were measured separately for moderate and vigorous activity levels. Moderate activity included bowling, golf, light sports, or taking long walks, whereas vigorous physical activity included jogging, swimming, aerobics, or strenuous sports.

### Defining BMI trajectories

Latent Class Growth Mixture Modeling (LCGMM) [39] was used to define life-course BMI trajectories using PROC TRAJ [40] in SAS software version 9.4 [41]. This group-based trajectory modeling procedure identifies subgroups of people among the whole study population who share similar underlying trajectories of BMI over the life-course. Data on BMI from teenage years, young adulthood (20 s), mid-adulthood (30–40 s), and late adulthood (50–60 s) contributed to defining the trajectories. Current BMI was not used in the development of trajectories since we only had access to crud age groups and did not have exact current age due to privacy concerns.

Based on a priori knowledge [21, 42] we tested the fit of models with up to seven trajectories. Model fit statistics using the Bayesian information criterion (BIC) were used to identify the best number of trajectories and the significance of polynomial terms (linear, quadratic or cubic). Once the final model was determined, the posterior probabilities and percentage of group memberships were assessed to further assure the most optimal model was chosen. A 5-class model with a quadratic structure fit the data best. Names were assigned to each of the five trajectories based on visual inspection of the trajectory plots. Sensitivity analyses were conducted to evaluate trajectories among the controls only and a similar 5-class model fit best. Participants were assigned to a trajectory based on their posterior probability. The mean posterior probability for each group exceeded the recommended 0.70 cut-off [39]. Although it is recommended that each group should hold at least 5% group membership [43], we observed one group that consistently had < 5% of our sample and this group continued to emerge even when the number of classes was reduced. Upon visual inspection of the trajectory plots it was apparent that this group represented a unique class and was retained despite the small sample size.

### Statistical analysis

Descriptive analyses were reported including frequency and percent for cases and controls for all variables. All statistical analyses were conducted using SAS software 9.4 [44]. Unconditional multivariable logistic regression was used to estimate adjusted odds ratios (OR) with 95% confidence intervals (CI) for BMI at individual timepoints and BMI trajectories across the life-course and pancreatic cancer risk. Conditional logistic regression was not used since frequency matching was used (e.g., not individual matches), but matching factors were included in all analyses. Results are presented for three models: 1) a parsimonious model adjusted only for age and sex; 2) a fully adjusted model that included age, sex, and all potential confounders; and 3) a model that included age, sex, all potential confounders plus two potential mediators (diabetes and pancreatitis). All covariates were controlled for in the model as described in Table 1, which the exception of smoking status which was included in the model as ever/former/current, and alcohol consumption which was included as ever/never.

**Table 1:** Age group and sex-adjusted odds ratio estimates for pancreatic cancer risk factors among cases diagnosed in 2011-2013 and controls recruited in 2011, from Ontario, Canada

Characteristic	Cases (n = 310)		Controls (n = 1,258)		OR^a^	95% CI
	*n*	%	*n*	%		
Age (y)^b, c^						
< 60	79	26	444	35	–	–
60–64	65	21	282	20	–	–
65–69	58	19	220	17	–	–
≥ 70	104	34	312	25	–	–
Missing	4	1	0	0		
Gender^b^						
Male	164	53	662	53	–	–
Female	146	47	596	47	–	–
Education^d^						
College/university graduate	129	42	577	46	1.00	
High school graduate or less	180	58	680	54	1.14	0.88, 1.47
Missing	1	0.3	1	0.1		
Race						
Caucasian	265	85	1154	92	1.00	
Non-Caucasian	44	14	104	8	1.77	1.20, 2.60
Missing	1	0.3	0	0		
Family history of pancreas cancer^e^						
No	256	83	1144	91	1.00	
Yes	33	11	49	4	3.03	1.90, 4.83
Don’t know	20	7	61	5		
Missing	1	0.3	4	0.3		
Body mass index (kg/m^2^)^f^						
< 25.0	102	33	402	32	1.00	
25.0– < 30.0	109	35	519	41	0.86	0.63, 1.17
≥ 30.0	99	32	336	27	1.25	0.91, 1.73
Missing	0	0	1	0.1		
Alcohol intake^g^						
Never	107	35	406	33	1.00	
Former	27	9	85	7	1.26	0.77, 2.06
Current	173	56	756	60	0.82	0.59, 1.14
Light to moderate (1–20 drinks/wk)	138	45	658	54	0.83	0.57, 1.21
Heavy (21 + drinks/wk)	29	10	72	6	1.60	0.98, 2.64
Missing	3	1	11	1		
Cigarette smoking						
Never	118	38	573	46	1.00	
Ever	188	61	683	54	1.34	1.04, 1.74
> 0– < 8.5 pack years	59	19	223	18	1.29	0.91, 1.84
8.5– < 22 pack years	57	18	231	19	1.24	0.87, 1.77
≥ 22 pack years	66	21	220	18	1.46	1.03. 2.06
Missing	10	1	2	0.1		
Diabetes^h^						
No	247	80	1090	87	1.00	
Yes	63	20	159	13	1.61	1.16, 2.24
Missing	0	0	9	1		
Pancreatitis^h^						
No	290	94	1236	98	1.00	
Yes	20	6	14	1	5.72	2.82, 11.61
Missing	0	0	8	1		

^a^Age group and sex-adjusted OR

^b^No reported OR, controls frequency matched to cases

^c^Age at pancreas cancer diagnosis for cases; age at questionnaire completion for controls

^d^Highest level of education reached

^e^Immediate blood relatives

^f^One year prior to completion of questionnaire for both cases and controls

^g^Both cases and controls were asked to recall alcohol intake “approximately two years ago”

^h^Cases were asked about diagnosis “before one year ago”; controls were asked if ever diagnosed

Selected characteristics of the population-based controls participating in the OCRF study were compared to national population-based data from the 2011 Canadian Community Health Survey (CCHS) to determine how well the controls came from the source population as the cases. Survey data were used from the 2011 CCHS for adults aged 40 to 90 living in Ontario.

## Results

**Fig. 1:**
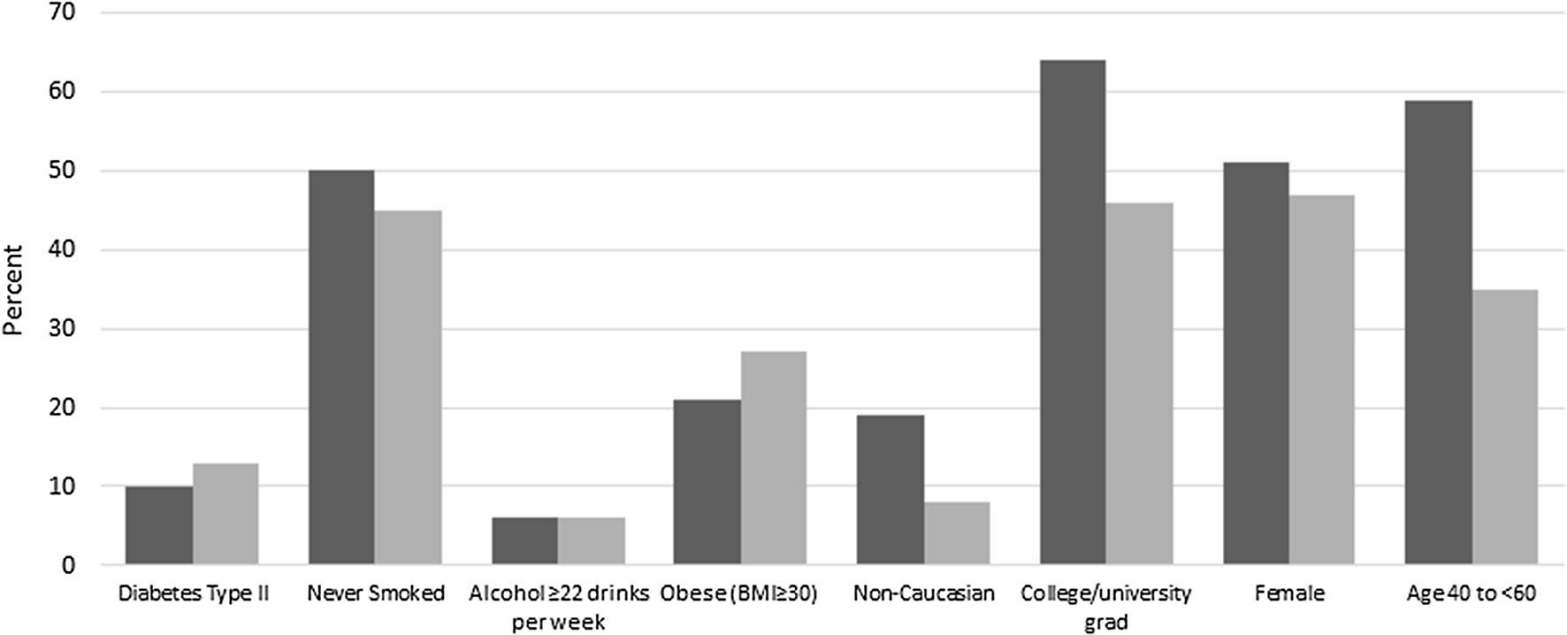
Representativeness of Ontario cancer risk factor (OCRF) study controls compared to the Canadian community health survey (CCHS) for Ontarians aged 40–90 in 2011. The dark bars represent the Canadian community health survey (CCHS) data, whereas, the light gray bars represent the Ontario cancer risk factor (OCRF) data

Table 1 provides the frequencies by case and control status and the age group and sex-adjusted OR for selected subject characteristics (some of which has been published previously [29, 45]). 53% of cases and controls were males, and 34% of cases and 25% of controls were over the age of 70. Majority of participants were Caucasian (91%), and more than half of both cases and controls had a highest education of a high school diploma or less. As shown previously in this study [29], family history of pancreas cancer, heavy alcohol consumption, cigarette smoking, current BMI ≥ 30.0, and non-Caucasian ethnicity were all associated with an increased odds of pancreatic cancer (see Table 1). Comparing the study data among controls only to the CCHS revealed that the data used in this study are representative of the general population of residents in Ontario (Fig. 1). The controls in our study had similar proportions for sex, diabetes, non-smokers, alcohol consumption patterns (> 22 drinks per week), however had slightly higher rates of obesity (BMI > 30). There were lower rates of non-Caucasian participants and those who attended post-secondary education among study controls in comparison to the provincial data. As expected, study controls were much older than the general population since they were frequency age-matched to cancer cases.

**Fig. 2:**
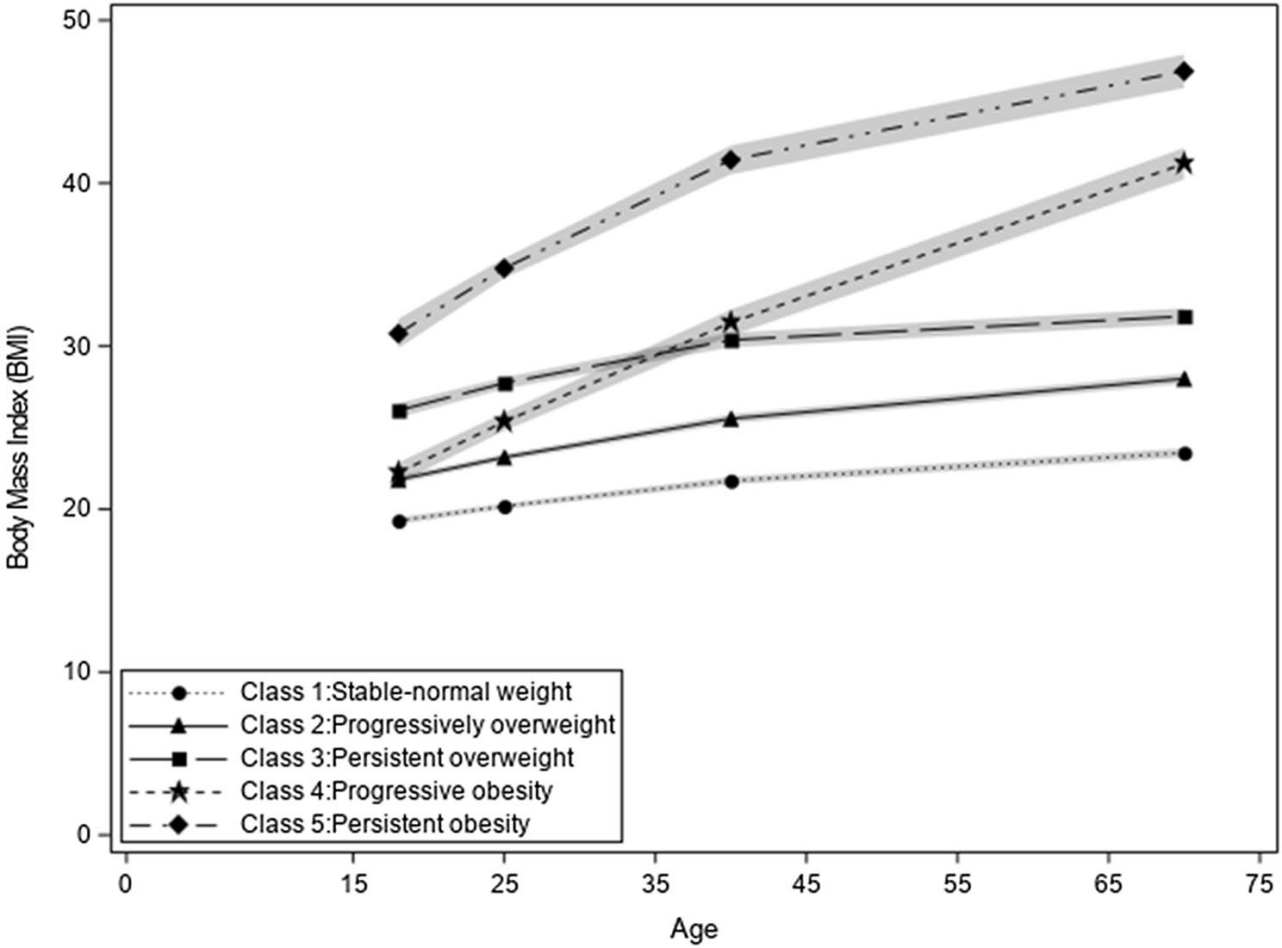
Latent BMI trajectories identified throughout the life-course (*n *= 1,568) among Cases Diagnosed in 2011–2013 and Controls Recruited in 2011, from Ontario, Canada. Class 1: Stable-normal weight trajectory is represented as the dotted line, with circular symbols. Class 2: Progressively overweigh trajectory is represented as the solid line, with triangular symbols. Class 3: Persistent overweight trajectory is represented as the line with long dashes, with square symbols. Class 4: Progressive obesity trajectory is represented as the line with small dashes, with star symbols. Class 5: Persistent obesity trajectory is represented as the line with dashes and dots, with diamond symbols

Five distinct trajectories of BMI across the life-course were identified (Fig. 2). The mean posterior probabilities were for groups 1–5 were 0.91, 0.87, 0.89, 0.90 and 0.97, respectively. We labeled these trajectories based on visual assessment; stable-normal weight (38.9%), progressively overweight (42.2%), persistent overweight (12.6%), progressive obesity (4.2%), and persistent obesity (2.1%). Generating the trajectories among the controls only revealed the same five distinct trajectories of BMI and with approximately the same proportion of individuals; stable-normal weight (37.3%), progressively overweight (43.0%), persistent overweight (12.8%), progressive obesity (4.5%), and persistent obesity (2.5%). Due to the small sample size in the persistent obesity trajectory a model with four trajectories was tested; however, the persistent obesity trajectory remained with a very small sample size. This trajectory consistently emerged throughout the various stages of model building.

Characteristics of the participants in each BMI trajectory among controls only are described in Table 2. More than half of the participants in the stable-normal weight trajectory (63%) and persistent obesity trajectory (71%) were female. Consistent with the trajectories created including both cases and controls, the persistent obesity trajectory among controls only were considerably younger than other trajectories (74% < 60 years of age). The progressive obesity trajectory had the greatest proportion of participants educated to high school or less (70%), whereas the remaining trajectories had about half educated to this level. Across all trajectories, the proportion of participants who were an ever smoker was about half. For current alcohol consumption, the persistent obesity trajectory had the smallest proportion (29%), whereas the remaining trajectories had proportions ranging from 48 to 62%.

**Table 2:** Descriptive characteristics of the bmi trajectory groups among controls only, recruited in 2011, from Ontario, Canada (*n* = 1,258)

Characteristics	Class 1: stable-normal weight (*n* = 469) %	Class 2: progressively overweight (*n* = 541) %	Class 3: persistent overweight (*n* = 161) %	Class 4: progressive obesity (*n* = 56) %	Class 5: persistent obesity (*n* = 31) %
Age (y)^a^					
< 60	33	33	41	45	74
60–64	21	23	24	27	26
65–69	18	18	21	14	0
≥ 70	29	27	15	14	0
Sex					
Female	63	39	26	54	71
Male	37	61	74	46	29
Education^b^					
High school graduate or less	51	54	57	70	52
College/university graduate	49	46	43	30	48
Race					
Non-Caucasian	12	7	4	4	10
Caucasian	88	93	94	96	90
Young adulthood (20 s) BMI					
< 25.0	99	79	6	43	0
25.0– < 30.0	1	20	78	50	10
≥ 30.0	0	0	15	4	87
Mid-adulthood (30–40 s) BMI					
< 25.0	98	43	1	9	0
25.0– < 30.0	1	53	53	20	0
≥ 30.0	0	2	45	70	97
Late adulthood (50–60 s) BMI					
< 25.0	69	9	1	0	0
25.0– < 30.0	18	64	18	0	0
≥ 30.0	0	12	65	82	68
Age not reached	12	11	16	18	32
Alcohol intake^c^					
Current	62	61	60	48	29
Former/never	38	39	40	52	71
Smoking status					
Ever	53	55	56	59	54
Never	47	45	44	41	46
Diabetes^d^					
No	93	86	83	68	68
Yes	7	13	17	30	32
Missing	1	1	0.6	2	0
Pancreatitis^d^					
No	98	99	97	96	100
Yes	1	1	2	4	0
Missing	1	0.4	1	0	0

^a^Age at pancreas cancer diagnosis for cases; age at questionnaire completion for control

^b^Highest level of education reached

^c^Both cases and controls were asked to recall alcohol intake “approximately two years ago”

^d^Cases asked about diagnosis “before one year ago”; controls asked if ever diagnosed

The association between each distinct trajectory and pancreatic cancer risk is reported in Table 3. Compared to the stable-normal weight trajectory (reference group), the progressively overweight (adjusted OR: 1.17; 95% CI 0.86, 1.60), persistently overweight (adjusted OR: 1.72; 95% CI 1.12, 2.64) and progressive obesity (adjusted OR: 1.70; 95% CI 0.88, 3.30) all had increased odds of pancreatic cancer. The persistent obesity trajectory was inversely associated with odds of pancreatic cancer (adjusted OR: 0.24; 95% CI 0.03, 1.79), but with a very wide confidence interval which limits interpretation of these results. The models adjusted for all potential confounders identified a priori (Model 2) were not substantially different from the models adjusted for age and sex only. Further adjustment for diabetes and pancreatitis (potential mediating variables) in Model 3 did not yield results that were substantially different from the fully adjusted ORs (Table 3). Sensitivity analyses were conducted removing the last timepoint for all participants in case of weight loss due to disease. When the last time point was removed five trajectories were identified that were very similar to the first three timepoints in the main results and the associations with pancreatic cancer were similar.

**Table 3:** Odds ratio estimates for latent BMI trajectory classes and pancreatic cancer among cases diagnosed in 2011–2013 and controls recruited in 2011, from Ontario, Canada

Trajectory classes	Cases		Controls		Model 1		Model 2		Model 3	
	*n* = 310	%	*n *= 1,258	%	OR^a^	95% CI	OR^b^	95% CI	OR^c^	95% CI
Class 1: stable-normal weight	117	38	493	39	1.00		1.00		1.00	
Class 2: progressively overweight	128	41	534	43	1.07	0.81, 1.43	1.17	0.86, 1.60	1.15	0.84, 1.58
Class 3: persistent overweight	349	16	149	12	1.55	1.04, 1.32	1.72	1.12, 2.64	1.63	1.05, 2.53
Class 4: progressive obesity	14	5	51	4	1.35	0.72, 2.54	1.70	0.88, 3.30	1.51	0.77, 2.99
Class 5: persistent obesity	2	0.7	31	3	0.36	0.08, 1.53	0.24	0.03, 1.79	0.22	0.03, 1.70

^a^Adjusted for age group and sex

^b^Adjusted for age group, sex, race, alcohol consumption, smoking, vegetable consumption, fruit consumption, red meat consumption, current moderate physical activity, current vigorous physical activity, family history of pancreatic cancer

^c^Adjusted for all variables in Model 2 plus pancreatitis, diabetes

The associations between overweight and obesity at each time period over the life-course and pancreatic cancer risk are reported in Table 4. Obesity during young adulthood (adjusted OR: 1.29; 95% CI 0.69, 2.43), and both overweight (OR: 1.27; 95% CI 0.93, 1.74) and obesity during mid-adulthood (OR: 1.49; 95% CI 0.98, 2.28) were consistently associated with increased odds of pancreatic cancer in the minimally adjusted and fully adjusted models but were not statistically significantly different from 1.0. Conversely, overweight and obesity during later adulthood did not appear to be strongly associated with odds of pancreatic cancer. Although the sample size was small, and the confidence intervals were wide, these findings may possibly suggest that increased BMI in early life is a stronger risk factor for pancreatic cancer than exposure during later adulthood.

**Table 4:** Odds ratio estimates for recalled body mass index (BMI) by age period and pancreatic cancer among cases diagnosed in 2011-2013 and controls recruited in 2011, from Ontario, Canada

Body mass index (kg/m^2^) for various time periods	Cases (*n* = 310) N	%	Controls (*n *= 1,258) N	%	Model 1		Model 2		Model 3	
					OR^a^	95% CI	OR^b^	95% CI	OR^c^	95% CI
Adolescent^d^										
< 25.0	246	80	1052	84	1.00		1.00		1.00	
≥ 25.0	49	16	188	15	1.13	0.81, 1.63	1.18	0.81, 1.72	1.16	0.79, 1.71
Missing	15	4	18	1						
Young adulthood (20 s)										
< 25.0	229	74	927	74	1.00		1.00		1.00	0.70, 1.42
25.0– < 30.0	61	20	266	21	0.97	0.70, 1.35	0.96	0.68, 1.36	0.99	0.70, 1.42
≥30.0	17	5	53	4	1.47	0.83, 2.61	1.29	0.69, 2.43	1.11	0.58, 2.15
Missing	3	1	12	1						
Mid-adulthood (30–40 s)										
< 25.0	161	52	700	56	1.00		1.00		1.00	
25.0– < 30.0	103	33	390	31	1.24	0.93, 1.66	1.27	0.93, 1.74	1.23	0.89, 1.68
≥ 30.0	43	14	155	12	1.39	0.94, 2.06	1.50	0.98, 2.28	1.39	0.90, 2.14
Missing	3	1	4	0.3						
Late adulthood (50–60 s)										
< 25.0	103	33	374	30	1.00		1.00		1.00	
25.0– < 30.0	109	35	461	37	0.63	0.36, 1.12	0.91	0.65, 1.27	0.89	0.63, 1.25
≥ 30.0	75	24	255	20	0.91	0.67, 1.24	1.20	0.82, 1.76	1.10	0.74, 1.64
Age not reached	21	7	163	13						
Missing	2	0.6	1	0.8						

^a^Adjusted for age group and sex

^b^Adjusted for age group, sex, race, alcohol consumption, smoking, vegetable consumption, fruit consumption, red meat consumption, current moderate physical activity, current vigorous physical activity, family history of pancreatic cancer

^c^Adjusted for the variables in Model 2 with the addition of pancreatitis, diabetes

^d^BMI categories 25.0– < 30.0 and ≥ 30.0 were collapsed for the adolescent time period due to small cell sizes when BMI ≥ 30.0 was evaluated alone

A stratified analysis was conducted by sex to evaluate possible effect modification. In the analyses stratified by sex, the persistent overweight trajectory, in comparison to the stable-normal trajectory, was associated with increased odds of pancreatic cancer in in males (OR: 2.58; 95% CI 1.45, 4.47) only, but not in females (OR: 0.55; 95% CI 0.21, 1.42) (Online Resource 1; Table 1). The progressively overweight and the progressive obesity trajectories had ORs greater than 1.0 in both males and females, possibly suggestive of increased odds of pancreatic cancer relative to the stable-normal weight trajectory; however, the results had very wide confidence intervals that overlapped 1.0 (Online Resource 1; Table 1). The persistent obesity trajectory results were hard to interpret separately in both males and females due to very small cell sizes and wide confidence intervals. When the results for BMI at each of the specific age periods were stratified by sex (Online Resource 1; Table 2), it appeared that the increased odds of pancreatic cancer associated with higher BMI was only present for males, but not females. Males with BMI > 30 at each age period had increased risk of pancreatic cancer, this was the strongest for young adulthood (OR: 2.69; 95% CI 1.22, 5.90) and mid-adulthood (OR: 2.30; 95% CI 1.32, 4.01). Among females, all of the estimates had wide confidence intervals (overlapping 1.0) but many of the OR were less than 1.0 which is inconsistent with the hypothesis that overweight and obesity are associated with pancreas cancer risk.

## Discussion

The results of this study contribute to the growing body of literature that describe life-course BMI trajectories and the association with pancreatic cancer. When BMI during each period of life was evaluated separately, the strongest associations with pancreatic cancer emerged in young and mid-adulthood. Further, five distinct trajectories of BMI across the life-course were identified and the persistent overweight and progressive obesity trajectories had ORs greater than 1.0 suggestive of increased risk. It is interesting that the magnitude of the ORs were greatest in the persistent overweight category which was characterized by higher BMI in the teenage years. The results for the persistent obesity trajectory, characterized by the highest BMI across the life-course, were somewhat unexpected in that the OR for the association with pancreatic cancer was less than 1.0; however, the number of participants in this trajectory was small (2%) and the 95% CI was very wide. Evaluation of the characteristics associated with each trajectory among controls only showed that individuals in the persistent obesity trajectory were less likely to be in the older age group. Even though we adjusted for age group as a potential confounder (data on exact age were not accessible due to privacy regulations), it is possible that there is unmeasured or residual confounding by age. It may also be possible individuals in the persistent obesity group are less likely to be diagnosed with pancreatic cancer, due to earlier mortality from other diseases such as cardiovascular disease.

The five distinct life-course BMI trajectories that we identified are consistent with previous studies of similar methodologies. The Nurses’ Health and Health Professionals Follow-up Study [21] and the Young Finns Study [42] both identified five trajectories describing body shape/BMI. However, the Young Finns Study identified a resolving obesity trajectory where the participants became a healthy BMI later in life. These studies include data prior to the teenage years unlike the data included within our study. Both studies, along with our findings identified the persistent obesity trajectory to have the smallest sample size. Understanding the life-course trajectories is important, as in our study population four of the five identified trajectories led to overweight or obesity in later adulthood. However, each revealed very different patterns of how overweight and obesity developed. These details would not be perceptible in studies that only evaluate exposure in older life. Further, in our descriptive analyses of characteristics across trajectories it is apparent that there are unique distinctions.

Few studies have evaluated the effect of BMI before age 18 [11, 20] or life-course trajectories of body shape [21] and pancreatic cancer risk. Consistent with our findings, studies have identified an increased risk of pancreatic cancer in people who were overweight or obese during their adolescence [18, 20, 46–48]. Specifically, a prospective cohort study that followed participants for an average of 23 years, found men and women who were obese during ages 16–19 to be more likely to develop pancreatic cancer (HR: 3.67; 95% CI 2.52, 5.34 for men; HR:4.07; 95% CI 1.78, 9.29 for women) [20]. There is also evidence supporting an association between people with overweight and obesity in adulthood and pancreatic cancer risk [11, 12, 47, 48]. A pooled analysis [48] of 14-cohort studies in the Japanese population found an association between men aged 40 and above with obesity and pancreatic cancer (HR: 1.71; 95% CI 1.03–2.86). A systematic review [14] that evaluated BMI and incidence of cancer found higher BMI in adults to increase pancreatic cancer risk from 16 prospective datasets (RR:1.07 95% CI 0.93, 1.23 per 5 kg/m^2^ in men; RR:1.12; 95% CI 1.03, 1.23 per 5 kg/m^2^ in women). Some results in our study are consistent with these, as we identified ORs ranging from 1.20 to 1.50 for participants with overweight and obesity during the 30–40 s, but our results did not provide evidence of an association between overweight and obesity in the 50–60 s and pancreatic cancer risk.

Song et al., studied trajectories of body shape across the life-course and the risk of pancreatic cancer. It was noted that those who were in the heavy-stable and increasingly heavy body shape trajectory had an increased risk of pancreatic cancer (RR: 1.39; 95% CI 0.91, 2.12) [21] in comparison to those who were lean-stable throughout the life-course. These results are consistent with what we have found, as those who were in the progressive obesity group had an increased risk of pancreatic cancer (OR: 1.49; 95% CI 0.77, 2.87). Levi et al. found an increased risk of pancreatic cancer in individuals who were overweight in adolescence using Cox proportional hazards modeling (HR: 2.09; 95% CI 1.26, 3.50) [20], which also aligns with our study findings.

Our study had limited statistical power to evaluate subgroups, however, the results of our stratified analysis by sex suggest that there may be differences in both the BMI trajectories and age-specific BMI categories in males and females. While strong increased associations with earlier life overweight and obesity were consistently observed among males, these associations were not observed among females. This is not consistent with one previous study that evaluated life-course BMI trajectories and pancreatic cancer risk [21] and previous reviews of later adult BMI and pancreas cancer have not reported sex differences [12]. It is possible that our results may be due to measurement error or information bias if females recall early life BMI differently than males. It is a limitation of our study that BMI was collected based on self-reported recall of early life weight. However, in epidemiologic research, using self-reported recall of BMI may be the most feasible option. Few historical records of weight exist limiting the potential of retrospective cohort studies and it is often not feasible to conduct a prospective cohort study from early life to late adulthood [49, 50]. Several studies have investigated the validity of self-reported recall of early life BMI compared to prospective measurement or historical records, and relatively high correlation has been found (r = 0.74–0.84) [32, 33, 37, 51]. A recent systematic review found self-reported recall of past weight and prospectively measured weight had a small mean difference (0.87 kg; 95% CI 0.19–1.56) and was highly correlated (pooled r = 0.83; 95% CI 0.72–0.90). [30] There were small mean differences reported across studies that assessed the validity of self-reported recall and reference measures of BMI [32, 33, 37, 51, 52]. Must et al. [33] found males underreported past BMI by 0.4 kg/m^2^ and females underreported past BMI by 1.30 kg/m^2^ across a mean period of recall of 55 years. Although self-reported recall of early life BMI or body fatness may be a valid measure, there are always limitations and potential biases from the use of BMI as a measure of obesity [53]. A limitation of this study may also be the broad age ranges for which participants were asked to recall their BMI, the questionnaires did not ask participants to report for exact ages. Since the questionnaire that was used to capture this information did not specify the exact years of age for this period, participants may have recalled their body size for different ages due to the broad nature of the question. Inadequate data on date of diabetes and pancreatitis diagnosis and duration of disease are another limitation of this study. Future research may further explore the impact of these factors on life-course BMI trajectories and risk of pancreatic cancer.

Strengths of this study include the population-based sampling strategy used to recruit cases and controls. The detailed nature of the questionnaire allowed for a comprehensive assessment of weight across the life-course and a wide range of potential confounders, including smoking and physical activity. Although we comprehensively assessed known pancreatic cancer risk factors, there still may be residual confounding due to measurement error or by other unmeasured confounders. Further, while we assessed BMI across four time periods, we did not have available data on early life BMI (prior to teenage years) which may limit the findings of this study. Without this data, evaluating a sensitive period of growth and development that impact risk of pancreatic cancer may be limited.

As with all case–control studies, a potential limitation of this study is recall bias. Survival bias may be a concern with this study since the outcome of interest is a high fatality disease. Every effort was made to reduce survival bias by recruiting cases shortly after diagnosis through the Ontario Cancer Registry’s rapid-case ascertainment system. However, we can not rule out the possibility of survival bias, particularly with respect to the inconsistent findings for the persistent obesity trajectory if the cases with persistent obesity who participated in this study are systematically different from other adults with persistent obesity. While the low response rate and possibility of sampling bias may also threaten study validity, it was observed that most established pancreas cancer risk factors were associated with disease in the expected direction. Future studies would benefit from a larger sample size with additional statistical power to evaluate effect modification and potential interactions of sex, and possibly other factors such as smoking status, with each BMI trajectory.

The results from this study suggest BMI trajectories across the life-course are differentially associated with pancreatic cancer risk. Understanding the cumulative effect of BMI across the life-course may inform early life obesity prevention in turn reducing the burden of disease associated with pancreatic cancer.

## Electronic supplementary material

Below is the link to the electronic supplementary material.

Supplementary material 1 (DOCX 18 kb)

## Data Availability

Data are available from the Ontario Pancreas Cancer Study and Ontario Cancer Risk Factor Study; however, access restrictions apply (data transfer agreement required by Cancer Care Ontario, and REB approval would be required). Authors Steven Gallinger and Michelle Cotterchio may be contacted for any requests at steven.gallinger@uhn.ca and michelle.cotterchio@cancercare.on.ca.
