# Supplementary material for: Trajectories of body mass index, from adolescence to older adulthood, and pancreatic cancer risk; a population-based case–control study in Ontario, Canada
Source: Cancer Causes Control. 2019 Jun 22;30(9):955–66. doi: 10.1007/s10552-019-01197-9 (PMC6685923; doi:10.1007/s10552-019-01197-9)
Supplement: Supplementary file 1 — Supplementary material 1 (DOCX 18 kb) [file 10552_2019_1197_MOESM1_ESM.docx]

**Trajectories of body mass index, from adolescence to older adulthood, and pancreatic cancer risk; a population-based case-control study in Ontario, Canada**

Vanessa De Rubeis^1^, Michelle Cotterchio^2,4^, Brendan T. Smith^3,4^, Lauren E. Griffith^1^, Ayelet Borgida^5^, Steven Gallinger^5,6^, Sean Cleary^7,8^, Laura N. Anderson^1, 9^

^1^Department of Health Research Methods, Evidence, and Impact, McMaster University, Hamilton, ON, Canada

^2^Prevention and Cancer Control, Cancer Care Ontario, Toronto, ON, Canada

^3^Public Health Ontario, Toronto, ON, Canada

^4^Dalla Lana School of Public Health, University of Toronto, Toronto, ON, Canada

^5^ Division of General Surgery. Toronto General Hospital, Toronto, ON, Canada

^5^ Lunenfeld-Tanenbaum Research Institute, Mount Sinai Hospital, Toronto, ON, Canada

^6^ Division of General Surgery. Toronto General Hospital, Toronto, ON, Canada

^7^Department of Surgery, University Health Network, University of Toronto, Toronto, ON, Canada

^8^Division of Hepatobiliary and Pancreas Surgery, Mayo Clinic, Rochester, MN, USA

^9^Child Health Evaluative Sciences, The Hospital for Sick Children Research Institute, Toronto, ON, Canada

*Corresponding Author:* Laura N Anderson, McMaster University, 1280 Main Street West, Hamilton ON, Canada, L8S4L8, 905-525-9140 x 21725, [ln.anderson@mcmaster.ca](mailto:ln.anderson@mcmaster.ca)

**Table 1.** Odds Ratio Estimates for Latent BMI Trajectory Classes and Pancreatic Cancer among Cases Diagnosed in 2011-2013 and Controls Recruited in 2011, from Ontario, Canada, stratified by sex

| Trajectory Classes | **MALE** | | | **FEMALE** | | |
| --- | --- | --- | --- | --- | --- | --- |
|  | Cases  N=164  (%) | Controls  N=662  (%) | OR^a^  (95% CI) | Cases  N=146  (%) | Controls  N=596  (%) | OR^a^  (95% CI) |
| Class 1: stable-normal weight | 23 | 29 | 1.00 | 54 | 51 | 1.00 |
| Class 2: progressively overweight | 46 | 50 | 1.33 (0.83-2.16) | 35 | 34 | 1.08 (0.66-1.68) |
| Class 3: persistent overweight | 26 | 17 | 2.58 (1.45-4.47) | 5 | 7 | 0.55 (0.21-1.42) |
| Class 4: progressive obesity | 4 | 4 | 2.07 (0.76-5.62) | 5 | 5 | 1.43 (0.56-3.71) |
| Class 5: persistent obesity | 1 | 1 | NA^b^ | 1 | 4 | 0.29 (0.04-2.30) |

1. Adjusted for age group, sex, race, alcohol consumption, smoking, vegetable consumption, fruit consumption, red meat consumption, current moderate physical activity, current vigorous physical activity, family history of pancreatic cancer
2. OR could not be estimated due to small cell size

**Table 2.** Odds Ratio Estimates for Recalled Body Mass Index (BMI) by Age Period and Pancreatic Cancer among Cases Diagnosed in 2011-2013 and Controls Recruited in 2011, from Ontario, Canada, stratified by sex

| Body Mass Index (kg/m^2^) for various time periods | **MALES** | | | **FEMALES** | | |
| --- | --- | --- | --- | --- | --- | --- |
|  | Cases  N=164  (%) | Controls  N=662  (%) | OR^a^  (95% CI) | Cases  N=146  (%) | Controls  N=596  (%) | OR^a^  (95% CI) |
| Adolescent  <25.0  25.0-<30.0  ≥30.0 | 75  22  3 | 82  16  2 | 1.00  1.77 (1.10-2.85)  1.67 (0.47-5.93) | 93  7  0.7 | 88  9  3 | 1.00  0.64 (0.28-1.47)  0.32 (0.04-2.54) |
| Young adulthood (20s)  <25.0  25.0-<30.0  ≥30.0 | 61  31  9 | 65  31  4 | 1.00  1.23 (0.81-1.87)  2.69 (1.22-5.90) | 90  8  2 | 85  10  5 | 1.00  0.60 (0.28-1.26)  0.39 (0.11-1.44) |
| Mid adulthood (30s-40s)  <25.0  25.0-<30.0  ≥30.0 | 37  43  20 | 44  42  14 | 1.00  1.41 (0.92-2.17)  2.30 (1.32-4.01) | 70  23  8 | 70  19  11 | 1.00  1.20 (0.72-1.99)  0.76 (0.36-1.62) |
| Late adulthood (50s-60s)  <25.0  25.0-<30.0  ≥30.0  Age not reached | 25  40  28  7 | 24  44  20  12 | 1.00  0.89 (0.54-1.47)  1.70 (0.96-3.00) | 43  31  20  6 | 36  29  21  15 | 1.00  0.92 (0.56-1.50)  0.77 (0.43-1.37) |

1. Adjusted for age group, sex, race, alcohol consumption, smoking, vegetable consumption, fruit consumption, red meat consumption, current moderate physical activity, current vigorous physical activity, family history of pancreatic cancer
